# Clustering of the Metabolic Syndrome Components in Adolescence: Role of Visceral Fat

**DOI:** 10.1371/journal.pone.0082368

**Published:** 2013-12-20

**Authors:** Melkaye G. Melka, Michal Abrahamowicz, Gabriel T. Leonard, Michel Perron, Louis Richer, Suzanne Veillette, Daniel Gaudet, Tomáš Paus, Zdenka Pausova

**Affiliations:** 1 The Hospital for Sick Children, University of Toronto, Toronto, Ontario, Canada; 2 Department of Epidemiology, Biostatistics and Occupational Health, McGill University, Montreal, Quebec, Canada; 3 Montreal Neurological Institute, McGill University, Montreal, Quebec, Canada; 4 ÉCOBES, Recherche et transfert, Cégep de Jonquière, Jonquière, Quebec, Canada; 5 Department of Human Sciences, Université du Québec à Chicoutimi, Chicoutimi, Quebec, Canada; 6 Department of Psychology, Université du Québec à Chicoutimi, Chicoutimi, Quebec, Canada; 7 Community Genomic Centre, Université de Montréal, Chicoutimi, Quebec, Canada; 8 Rotman Research Institute, University of Toronto, Toronto, Ontario, Canada; Wake Forest University Health Sciences, United States of America

## Abstract

Visceral fat (VF) promotes the development of metabolic syndrome (MetS), which emerges as early as in adolescence. The clustering of MetS components suggests shared etiologies, but these are largely unknown and may vary between males and females. Here, we investigated the latent structure of pre-clinical MetS in a community-based sample of 286 male and 312 female adolescents, assessing their abdominal adiposity (VF) directly with magnetic resonance imaging. Principal component analysis of the five MetS-defining variables (VF, blood pressure [BP], fasting serum triglycerides, HDL-cholesterol and glucose) identified two independent components in both males and females. The first component was sex-similar; it explained >30% of variance and was loaded by all but BP variables. The second component explained >20% of variance; it was loaded by BP similarly in both sexes but additional loading by metabolic variables was sex-specific. This sex-specificity was not detected in analyses that used waist circumference instead of VF. In adolescence, MetS-defining variables cluster into at least two sub-syndromes: (1) sex-similar metabolic abnormalities of obesity-induced insulin resistance and (2) sex-specific metabolic abnormalities associated with BP elevation. These results suggest that the etiology of MetS may involve more than one pathway and that some of the pathways may differ between males and females. Further, the sex-specific metabolic abnormalities associated with BP elevation suggest the need for sex-specific prevention and treatment strategies of MetS.

## Introduction

Metabolic syndrome (MetS) is defined as a cluster of risk factors for cardiovascular disease (CVD) and type-2 diabetes mellitus (T2DM) occurring in the same individual; it includes elevated blood pressure (BP), atherogenic dyslipidemia (raised triglycerides [TG] and lowered HDL-cholesterol [HDL-chol]), raised fasting glucose (Glu) and abdominal obesity. For diagnostic purposes, MetS is defined by the presence of at least three out of these five risk factors [1]. The syndrome is associated with a two-fold increase for the risk of CVD and a five-fold increase for the risk of T2DM [2]. Therefore, it is alarming to see that the prevalence of MetS is reaching epidemic proportions worldwide. In Canada and USA, for example, >25% of adults suffer from the syndrome [3], [4], [5]. Moreover, MetS, typically regarded as a middle- to late-adulthood disorder, is now emerging in adolescence [6] with close to 10% of all 12–19-year olds being affected [7], [8]. This emergence of MetS in adolescence has been in part attributed to obesity; it is one of the components as well as the main risk factor for MetS, and its prevalence has tripled during the last 30 year in this age group [9], [10].

Obesity-related risk for MetS increases not only with the quantity of body fat but also with its distribution – individuals who store body fat viscerally rather than elsewhere in the body are at a greater risk [11], [12], [13]. Abdominal obesity is typically assessed with a readily available waist circumference (WC), that reflects not only the quantity of fat but also the quantity of lean body mass (muscles, bones and internal organs) and cannot distinguish between subcutaneous and visceral fat. As such, WC may misclassify visceral obesity: there are individuals who have a normal WC but an excessive amount of VF and high risk for MetS, and individuals who have a large WC but a normal amount of VF and low risk for MetS [14].

Despite the critical role of VF in MetS pathogenesis and the recent emergence of MetS in adolescence, only a few large-scale population-based studies quantified VF directly (with magnetic resonance imaging [MRI] or computed tomography) [11], [12], [15]; none of them were conducted in adolescents. Furthermore, sex differences exist in the prevalence of some MetS components and in certain relationships between MetS-defining variables. For example, hypertension is more common in men than women [16] and VF is more closely correlated with BP in males than females [11], [17], [18]. Therefore, the aim of the present study was to examine the clustering of VF (measured directly using MRI) with other MetS-defining variables in community-based samples of males (n = 286) and females (n = 312) during adolescence, when the initial stages of MetS (“pre-clinical” disease [19]) may be emerging.

## Methods

### Ethics Statement

Written consent of the parents and assent of the adolescents were obtained before the commencement of data collection. The Research Ethics Committee of the Chicoutimi Hospital and the Hospital for Sick Children in Toronto approved the study protocol.

### Participants

Caucasian male (n = 286) and female (n = 312) adolescents aged 12 to 18 years were recruited from the French-Canadian population living in the Saguenay-Lac St. Jean region of Quebec, Canada, as part of the Saguenay Youth Study (SYS). The SYS is a population-based, cross-sectional study of cardiovascular, metabolic and brain health in adolescence. All participants were recruited through local high schools, as we described previously [20]. The current sample consists of participants recruited and tested between November 2003 and June 2009.

### Assessments

All participants underwent a 15-hour cardiovascular, metabolic and brain phenotyping protocol, which included assessments of all five MetS-defining variables [20].

Abdominal obesity was assessed with MRI and by measuring WC. MRI was used to measure volume of VF. MRI is currently the only non-invasive (i.e. without radiation) method that can measure VF directly in population-based studies of children and adolescents [21], [22]. T1-relaxation time for adipose tissue is much shorter than that of most other tissues, and thus T1-weighted MRI produces images on which fat is very bright and easy to segment by semi-automated or automated techniques [23]. In the present study, volume of VF was measured with a semi-automated technique [24] from a 10-mm-thick (in-plane resolution 1.56×1.56 mm^2^) axial T1-weighted image (repetition time/echo time = 200 ms/20 ms) acquired at the level of the umbilicus on a Phillips 1.0-T magnetic resonance scanner. In more detail, images were smoothed using an adaptive bilateral filter to remove image noise while preserving edge information. An initial fat-classification map was obtained using a standard region-growing algorithm. An iterative refinement procedure corrected false positives and false negatives using a battery of morphological operators, including hysteresis, thresholding over small neighborhoods and median filtering to remove salt and pepper noise. The resulting classification map was manually segmented into VF, which was defined as adipose tissue lying within the innermost aspect of the abdominal cavity, and not contained within other abdominal organs or muscles. A histogram counting algorithm computed the total number of voxels for VF. This semi-automated method was validated against manual segmentation in 20 randomly selected subjects (r^2^ = 0.97), as described previously [17]. In addition, height and weight were measured using standard operating procedures [20] and BMI was calculated as weight (in kilograms) divided by height (in meters) squared.

BP was measured beat-by-beat during a 52-min cardiovascular protocol designed to mimic daily life activities, such as changes in posture and mental stress. This protocol included a succession of three 10-min periods when participants were in supine, standing and sitting positions, which was followed by a 2-min math-stress and 10-min stress-recovery periods (both in a sitting position). The recording was made with a non-invasive hemodynamic monitor Finometer™ (FMS Finapres, Amsterdam, The Netherlands); the device measures finger blood-flow continuously and, from these data, it derives beat-by-beat brachial BP using the reconstruction and level-correction of the finger blood-flow waveform. The Finometer™ is a reliable device for tracking BP in adults and children older than six years [25]. Averages of systolic BP (SBP) during the five sections of the cardiovascular protocol described above (supine, standing, sitting, mental stress and mental stress recovery) were used for statistical analyses. We chose to study SBP because (1) systolic rather then diastolic hypertension is predominant among obese children [26] and young adults [27], and (2) population variance in SBP vastly exceeds that in diastolic BP [28].

Serum concentrations of TG, HDL-chol and Glu were measured from a blood sample drawn between 8 AM and 9 AM after overnight fasting; the measurements were made at the clinical Biochemistry Department of the Hôtel-Dieu Hospital (Montreal, Quebec, Canada).

In the present study, we defined MetS according to the recommendations of the International Diabetes Federation; the panel proposes that, for age 10 to <16 years, adult criteria are used for BP, TG, HDL-chol and Glu and age-specific criteria are used for waist circumference [29], and, for age ≥16 years, adult criteria are used for all components of MetS [1], [6]. The exact cut-off values are provided in Table S1. The prevalence of MetS was 2.9% in males and 1.3% in females (Table S1), which is comparable to that reported previously [7].

### Statistical Methods

Descriptive statistics used to characterize the study population included means and standard deviations (SDs) for continuous variables and proportions for categorical variables (presented in Table 1). Our main analyses focused on examining the architecture of pre-clinical MetS by identifying components of shared variance using principal component analysis (PCA) of the five MetS-defining variables, namely SBP, fasting serum TG, HDL-chol, Glu and VF. We also examined whether the components of shared variance differ when VF is replaced with WC. Prior to PCA, we assessed the normality assumption on which the statistical inference about PCA relies. VF, WC and serum TG, HDL-chol and Glu had positively skewed distributions and were log transformed using logarithm with base 10, which improved the fit. Before conducting PCA, all variables were adjusted for age and, in the case of VF, WC and SBP also for height. Age and height are known to correlate with BP in children and adolescents [30], [31].

**Table 1:** Basic characteristics and main outcomes in studied adolescent males and females.

Characteristic/Outcome	Males		Females		P-value
	N	Mean±SD/N	N	Mean±SD/N	
Age (years)	286	15.0±1.8	310	15.1±1.9	0.37
Height (cm)	281	167.0±10.6	306	160.1±6.6	<0.001
Weight (kg)	274	59.0±13.8	307	54.8±10.6	<0.001
BMI (kg/m^2^)	276	21.2±3.8	306	21.4±3.6	0.64
BMI (log kg/m^2^)	276	1.32±0.07	306	1.32±0.07	0.60
Visceral fat (cm^3^)*	276	27.1±28.4	296	23.4±16.9	0.70
Visceral fat (log cm^3^)	276	1.25±0.38	296	1.26±0.29	0.60
Waist circumference (cm)*	273	72.8±8.9	306	69.4±8.1	<0.001
Waist circumference (log cm)	273	1.86±0.05	306	1.84±0.05	<0.001
Supine SBP (mm Hg)	236	118.9±11.5	263	118.2±10.2	0.50
Standing SBP (mm Hg)	233	125.1±13.7	259	120.1±12.2	<0.001
Sitting SBP (mm Hg)	237	125.4±13.6	262	120.2±11.9	<0.001
Math SBP (mm Hg)	235	140.3±16.8	260	133.3±15.1	<0.001
Post-math SBP (mm Hg)	234	128.0±12.6	259	123.8±12.2	<0.001
Triglycerides (mmol/L)*	253	1.02±0.46	277	1.07±0.45	0.12
Triglycerides (log mmol/L)	253	−0.03±0.19	277	−0.01±0.18	0.16
HDL-cholesterol (mmol/L)*	254	1.40±0.28	277	1.56±0.32	<0.001
HDL-cholesterol (log mmol/L)	254	0.14±0.09	277	0.18±0.09	<0.001
Glucose (mmol/L)*	254	4.80±0.42	275	4.64±0.43	<0.001
Glucose (log mmol/L)	254	0.68±0.04	275	0.66±0.04	<0.001
Fasting insulin (pmol/L)	243	67.6±30.5	266	78.4±28.8	<0.001

BMI: body-mass index. Non-adjusted means and standard deviations are presented. Sex differences were evaluated with Student T-test or with non-parametric Wilcoxon test when data were not normally distributed*.

PCA was used to identify components of shared variance among the five MetS-defining variables described above. To examine whether these components vary across the cardiovascular protocol, the analysis was repeated for each of the five sections protocol (i.e., supine, standing, sitting, stress and stress recovery). PCA is a multivariate statistical technique that transforms a number of possibly correlated variables into a number of uncorrelated variables, so called principal components [32]. Each principal component represents a different linear combination of the original correlated variables. The original variables are first normalized to their respective means and, then used to generate a correlation matrix (Tables S2 and S3). PCA is then performed by eigenvalue decomposition of the correlation matrix. Principal components with eigenvalue>1 and loadings of individual MetS variables ≥0.3 were considered significant, as suggested previously for data with sample size ≥100 [33]. These analyses were done in males and females both separately and together, in the latter case adjusting for sex (JMP, Release 9, SAS Institute Inc., Cary, NC).

## Results

**Figure 1:**
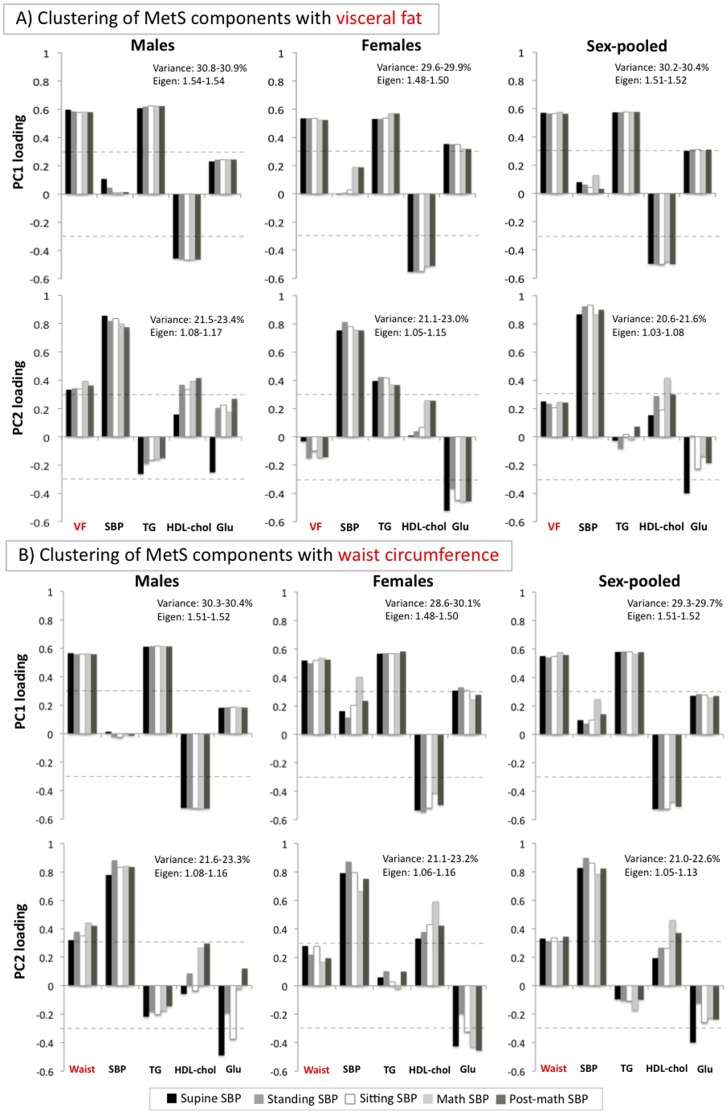
Clustering of MetS-defining variables with visceral fat or waist circumference. Principal component analysis (PCA) was performed with either visceral fat (VF) or waist circumference (WC) and remaining four MetS-defining variables: systolic blood pressure (SBP) and fasting serum concentrations of triglycerides (TG), HDL-cholesterol (HDL-chol) and glucose (Glu). To examine whether identified principal components vary across the cardiovascular protocol, the analysis was repeated for each of its five sections (i.e., supine, standing, sitting, stress and stress recovery). Principal components with eigenvalue>1 and loadings of individual MetS-defining variables ≥0.3 were considered significant [33]. These analyses were done in males and females separately and males and females together adjusting for sex (sex-pooled analyses). All variables were adjusted for age and when relevant for height prior to sex-separate PCA and additionally for sex prior to sex-pooled PCA.

PCA of the five MetS-defining variables (SBP, fasting serum TG, HDL-chol, Glu and VF) identified two independent components of shared variance in both males and females. The *first component*, PC1, explaining ∼30% of total variance, was almost identical in males and females. In both sexes, it was loaded by all MetS-defining variables except for SBP; VF, TG and Glu contributed positively and HDL-chol contributed negatively (Figure 1A). As pointed out above, this component did not involve SBP; this was the case across all five sections of the cardiovascular protocol (i.e., supine, standing, sitting, stress and post-stress) in both males and females (Figure 1A).

In contrast, the *second component*, PC2, explaining ∼20% of total variance, was loaded mainly by SBP in both sexes but, at the same time, SBP clustered with other MetS-defining variables differently in males and females. In males, SBP clustered positively with VF and HDL-chol. In females, SBP clustered positively with TG and negatively with Glu (Figure 1A). In both sexes, the clustering remained relatively constant throughout the cardiovascular protocol, except for the supine section when the contribution of HDL-chol in males was weaker (Figure 1A).

In most population-based studies, “abdominal obesity” is measured indirectly with WC. Therefore, we explored whether clustering of the five MetS-defining variables differs when WC is used instead of VF. In these analyses, the first component, PC1, remained similar, but the second component, PC2, changed (Figure 1B [WC]; compare with Figure 1A [VF]). In males, SBP no longer clustered with “abdominal obesity” and HDL-chol, but it did with “abdominal obesity” (represented here by WC) alone. In females, SBP no longer clustered with TG and Glu, but it did with HDL-chol and Glu (Figure 1B; compare with Figure 1A). Overall, sex differences of this component became less pronounced (Figure 1B; compare with Figure 1A).

Further, a potentially confounding effect of sex is frequently accounted for by adjusting statistically for sex. But this simple statistical treatment may not account for the existing biological differences between males and females [18]. To explore this issue, we performed sex-pooled analyses adjusted for sex, and compared them with the sex-specific analyses described above. These sex-pooled analyses showed that, as expected, the first component (PC1), which was similar in males and females in the sex-specific analyses, remained almost the same in the sex-pooled analysis (Figure 1A). But the second component (PC2), which was different in males and females in the sex-specific analyses, changed in that SBP did not cluster with any metabolic component (Figure 1A). This is likely due to the fact that the sex-specific metabolic correlates of SBP were often of the opposing relationship in males and females; for example, TG loaded positively in females but this variable loaded negatively in males (Figure 1A). In the pooled analyses, these sex-specific relationships canceled each other out.

## Discussion

The results of the present study demonstrate that the five MetS-defining variables cluster into at least two independent components of shared variance in both male and female adolescents. The first component, capturing the variance related to *obesity-related insulin resistance*, is similar in males and females, whereas the second component, capturing the variance associated with *metabolic correlates of BP*, is different in the two sexes. These results suggest that the etiology of MetS may involve more than one underlying pathway and that some of the pathways may differ in males and females.

In the present study, the first component of shared variance among the five MetS-defining variables was loaded positively by VF, TG and Glu and negatively by HDL-chol; BP did not load on this component at all. This component was the same in male and female adolescents. The loading of this component is consistent with the classical clustering of metabolic variables due to *obesity-induced insulin resistance*, whereby excess VF (a) increases peripheral and hepatic insulin resistance, which in turn augments fasting glycemia, and (b) promotes the development of atherosclerotic dyslipidemia, characterized by increased TG and decreased HDL-chol [1], [34]. In more detail, fat tissues secrete adipokines, such as TNFα and free fatty acids, which when released into the circulation enhance insulin resistance in skeletal muscle and liver [35]. Assuming some degree of failure of pancreatic β-cells, this leads to higher fasting glycemia. In addition, augmented lipid flux from fat tissues to the liver increases TG through augmented liver production of very-low density lipoproteins (VLDL), which are particles rich in TG. Increased TG, in turn, activate cholesteryl ester transfer protein, which is a plasma protein facilitating the transport of cholesteryl esters and TG between lipoproteins; it collects TG from VLDL and exchanges them for cholesteryl esters from HDL (and *vice versa*). This process results in TG enrichment of HDL and, at the same time, a decrease of cardio-protective HDL-chol [36]. As pointed out above, this cluster of *obesity-induced insulin resistance* was remarkably similar in males and females and remained virtually unchanged (a) when VF was replaced with WC as a measure of “abdominal obesity” and (b) when the two sexes were analyzed together and a potentially confounding effect of sex was accounted for by statistical adjustment for sex. These results suggest that the component is not specific to VF only and it is not influenced by sex. They are consistent with previous factor analyses of MetS in adults demonstrating the existence of sex-similar clustering of various obesity measures with insulin resistance and lipid abnormalities [37], [38].

In contrast, the second component of shared variance among the five MetS-defining variables was different in each sex and in sex-pooled analyses, and differed also when VF was replaced with WC as a measure of “abdominal obesity”. Although BP loaded into this component in all these analyses, the clustering of BP with additional metabolic variables varied. In males, BP clustered positively with VF and HDL-chol, whereas in females, it clustered positively with TG and negatively with Glu. In sex-pooled analyses, BP no longer clustered with any metabolic component, possibly due to the fact that the *sex-specific metabolic correlates of BP* were often of the opposing relationship with BP in males and females.

The sex-specific clustering of BP with MetS-defining metabolic variables suggests the existence of sex-specific mechanisms of BP regulation. This is consistent with the fact that BP is a sexually dimorphic trait, with both BP and the prevalence of hypertension being higher in males than females during reproductive age, beginning in early adolescence [39], [40]. In the present study, BP clustered positively with VF in males but not females. This sex difference in the relationship of VF to BP is not very well understood but it may, in part, be related to obesity-induced sympathoactivation. Sympathoactivation, which is one of the key mechanisms of BP elevation in obesity [41] is closely related to the quantity of VF [42] but not subcutaneous fat [43] and, in adolescence, VF-related sympathoactivation is seen in males but not females [17].

In the present study, BP also clustered with MetS-defining lipid variables (TG and HDL-chol) in a sex-specific manner. In females only, it clustered positively with TG, which is a finding consistent with studies demonstrating that acute administration of TG increases BP in animals and humans [44], [45]. In males only, it clustered positively with HDL-chol. Classically, high-density lipoproteins (HDL) have BP-lowering effects [46], [47]; however, in MetS and other cardiometabolic disease states, some of these protective properties of HDL may be lost due to structural changes of HDL [48], [49]. It is, perhaps, this fraction of so called “dysfunctional” HDL that contributes to the positive relationship between HDL-chol and BP observed in males.

Finally, BP also clustered with Glu in a sex-specific manner. The relationship was negative and was present only in females. Glu is an index of impaired glucose tolerance that develops due to insulin resistance (and pancreatic β-cell dysfunction). The inverse relationship between Glu and BP in females may result from the action of a molecule that *(a)* has both insulin-sensitizing (Glu-lowering) and fluid retentive (BP-elevating) effects and *(b)* shows higher expression/activity in females than males. One such molecule may be PPARγ: it has both insulin-sensitizing [50] and fluid-retentive [51], [52], [53] effects, and some of its actions are more pronounced in women than men [54]. PPARγ is a transcription factor expressed mainly in adipose tissue (but also in the kidneys) where it plays a key role in promoting adipogenesis as part of its insulin-sensitizing actions [55]. Enhanced adipogenesis, in turn, contributes to *accelerated weight gain* that occurs at later stages of pubertal development and is more pronounced in females than males [56]. Further experimental studies are required to support the possible role PPARγ in mediating the inverse relationship between BP and Glu and whether this relationship is specific to the later stages of puberty studied here (Table 1).

The current study is a cross-sectional investigation and, as such, no causal conclusions can be drawn. A longitudinal design would facilitate examination of causal relationships between, e.g., metabolic variables and BP, and their predictive value vis-à-vis the emergence of a full-blown MetS. Usefulness of cross-sectional studies, however, should not be underestimated, as they have generated many clinically highly relevant findings (e.g., NHANES III [7], Framingham Study [57] and Bogalusa Heart Study [58].

In summary, the results of the present study suggest that, in adolescence, MetS consists of at least two sub-syndromes, one reflecting sex-similar *metabolic abnormalities of obesity-induced insulin resistance* and the other capturing sex-specific *metabolic correlates of BP elevation.* The results also suggest that VF may enhance the first sub-syndrome (*obesity-induced insulin resistance*) in both males and females, whereas it may contribute to the second sub-syndrome (*BP elevation*) only in males. Adolescents may be an important age group for investigating the pre-clinical stages of MetS.

## Supporting Information

Table S1 **Prevalence of cardiometabolic risk factors in Saguenay Youth Study.**
(DOC)

Table S2 **Correlation matrix (visceral fat).**
(DOC)

Table S3 **Correlation matrix (waist circumference).**
(DOC)
