# Supplementary material for: Clustering of the Metabolic Syndrome Components in Adolescence: Role of Visceral Fat
Source: PLoS One. 2013 Dec 20;8(12):e82368. doi: 10.1371/journal.pone.0082368 (PMC3869691; doi:10.1371/journal.pone.0082368)
Supplement: Table S1 — Prevalence of cardiometabolic risk factors in Saguenay Youth Study. (DOC) [file pone.0082368.s001.doc]

**Table S1**: Prevalence of cardiometabolic risk factors in Saguenay Youth Study

| Component | Males | | Females | | p-value |
| --- | --- | --- | --- | --- | --- |
|  | n | n (%) | n | n (%) |  |
| “High” WC | 281 | 16 (5.7) | 310 | 20 (6.5) | 0.73 |
| “High” BP | 237 | 71 (30.0) | 260 | 40 (15.4) | 0.0001 |
| “High” TG | 255 | 27 (10.6) | 277 | 30 (10.8) | 1.0 |
| “Low” HDL-chol | 255 | 16 (6.3) | 277 | 60 (21.7) | <0.0001 |
| “High” Glu | 255 | 18 (7.1) | 277 | 11 (4.0) | 0.13 |
| MetS | 206 | 6 (2.9) | 230 | 3 (1.3) | 0.32 |

Serum concentrations of triglycerides (TG), high-density lipoprotein (HDL) cholesterol and glucose were measured from a fasting blood sample drawn between 8AM and 9AM. Metabolic syndrome (MetS) was defined according to the recommendations of the International Diabetes Federation and the presence of ≥3 of the following 5 conditions:

1. *“High.” WC*: waist circumference ≥ age- and sex-specific 90th percentile for 12- to <16-year old participants [1] and ≥102 cm for ≥16-year old male participants and ≥88 cm for v16-year old female participants [2].
2. *“High” BP*: systolic blood pressure ≥130 mm Hg, sitting, after 5 minutes of rest [2].
3. *“High” TG*: fasting serum concentration of triglycerides ≥1.7 mmol/L [2].
4. *“Low” HDL-chol*: fasting serum concentration of HDL-cholesterol <1.0 mmol/L for male participants and <1.3 mmol/L for female participants [2].
5. *“High” Glu:* fasting serum concentration of glucose ≥5.5 mmol/L [2].

Sex differences were evaluated with Fisher’s exact test.

**references**

1. Alberti KG, Eckel RH, Grundy SM, Zimmet PZ, Cleeman JI, Donato KA, Fruchart JC, James WP, Loria CM, Smith SC, Jr. (2009) Harmonizing the metabolic syndrome: a joint interim statement of the International Diabetes Federation Task Force on Epidemiology and Prevention; National Heart, Lung, and Blood Institute; American Heart Association; World Heart Federation; International Atherosclerosis Society; and International Association for the Study of Obesity. Circulation 120:1640-1645.
2. Wilson PW, D'Agostino RB, Parise H, Sullivan L, Meigs JB. (2005) Metabolic syndrome as a precursor of cardiovascular disease and type 2 diabetes mellitus. Circulation 112: 3066-3072.
