# Supplementary material for: Clustering of the Metabolic Syndrome Components in Adolescence: Role of Visceral Fat
Source: PLoS One. 2013 Dec 20;8(12):e82368. doi: 10.1371/journal.pone.0082368 (PMC3869691; doi:10.1371/journal.pone.0082368)
Supplement: Table S2 — Correlation matrix (visceral fat). (DOC) [file pone.0082368.s002.doc]

**Table S2:** Correlation matrix (visceral fat)

| **Males only** |  |  |  |  |  |
| --- | --- | --- | --- | --- | --- |
|  | VF | SBP | TG | HDL-chol | Glu |
| VF | 1.00 | 0.17 | 0.31 | -0.18 | 0.12 |
| SBP | 0.17 | 1.00 | -0.08 | 0.04 | -0.03 |
| TG | 0.31 | -0.08 | 1.00 | -0.26 | 0.14 |
| HDL-chol | -0.18 | 0.04 | -0.26 | 1.00 | 0.05 |
| Glu | 0.12 | -0.03 | 0.14 | 0.05 | 1.00 |
| **Females only** |  |  |  |  |  |
|  | VF | SBP | TG | HDL-chol | Glu |
| VF | 1 | 0.00 | 0.11 | -0.15 | 0.15 |
| SBP | 0.00 | 1.00 | 0.10 | 0.06 | -0.03 |
| TG | 0.11 | 0.10 | 1.00 | -0.26 | 0.03 |
| HDL-chol | -0.15 | 0.06 | -0.26 | 1.00 | -0.11 |
| Glu | 0.15 | -0.03 | 0.03 | -0.11 | 1.00 |
| **Sex-pooled** |  |  |  |  |  |
|  | VF | SBP | TG | HDL-chol | Glu |
| VF | 1 | 0.09 | 0.20 | -0.16 | 0.15 |
| SBP | 0.09 | 1.00 | 0.01 | 0.04 | -0.03 |
| TG | 0.20 | 0.01 | 1.00 | -0.25 | 0.09 |
| HDL-chol | -0.16 | 0.04 | -0.25 | 1.00 | -0.05 |
| Glu | 0.15 | -0.03 | 0.09 | -0.05 | 1.00 |

VF: visceral fat

SBP: sitting systolic blood pressure

TG: triglycerides

HDL-chol: HDL- cholesterol

Glu: glucose
