# Supplementary material for: Clustering of the Metabolic Syndrome Components in Adolescence: Role of Visceral Fat
Source: PLoS One. 2013 Dec 20;8(12):e82368. doi: 10.1371/journal.pone.0082368 (PMC3869691; doi:10.1371/journal.pone.0082368)
Supplement: Table S3 — Correlation matrix (waist circumference). (DOC) [file pone.0082368.s003.doc]

**Table S3:** Correlation matrix (waist circumference)

| **Males only** |  |  |  |  |  |
| --- | --- | --- | --- | --- | --- |
|  | Waist | SBP | TG | HDL-chol | Glu |
| Waist | 1.00 | 0.12 | 0.27 | -0.21 | 0.07 |
| SBP | 0.12 | 1.00 | -0.08 | 0.05 | -0.03 |
| TG | 0.27 | -0.08 | 1.00 | -0.26 | 0.14 |
| HDL-chol | -0.21 | 0.05 | -0.26 | 1.00 | 0.05 |
| Glu | 0.07 | -0.03 | 0.14 | 0.05 | 1.00 |
| **Females only** |  |  |  |  |  |
|  | Waist | SBP | TG | HDL-chol | Glu |
| Waist | 1.00 | 0.15 | -0.03 | 0.17 | -0.12 |
| SBP | 0.15 | 1.00 | -0.01 | 0.10 | 0.06 |
| TG | -0.03 | -0.01 | 1.00 | 0.08 | -0.14 |
| HDL-chol | 0.17 | 0.10 | 0.08 | 1.00 | -0.26 |
| Glu | -0.12 | 0.06 | -0.14 | -0.26 | 1.00 |
| **Sex-pooled** |  |  |  |  |  |
|  | Waist | SBP | TG | HDL-chol | Glu |
| Waist | 1.00 | 0.15 | 0.17 | -0.12 | 0.14 |
| SBP | 0.15 | 1.00 | 0.10 | 0.06 | -0.03 |
| TG | 0.17 | 0.10 | 1.00 | -0.26 | 0.03 |
| HDL-chol | -0.12 | 0.06 | -0.26 | 1.00 | -0.11 |
| Glu | 0.14 | -0.03 | 0.03 | -0.11 | 1.00 |

Waist: waist circumference

SBP: sitting systolic blood pressure

TG: triglycerides

HDL-chol: HDL- cholesterol

Glu: glucose
